# A Study to Evaluate Changes in Modified Mallampati Class in Patients Undergoing Spine Surgery in Prone Position

**DOI:** 10.7759/cureus.25767

**Published:** 2022-06-08

**Authors:** Mamta Jain, Jatin Lal, Diya Aggrawal, Jyoti Sharma, Anish K Singh, Teena Bansal

**Affiliations:** 1 Anesthesia, Pandit Bhagwat Dayal Sharma Post Graduate Institute of Medical Sciences, Rohtak, IND; 2 Anesthesia, All India Institute of Medical Sciences, Bathinda, IND

**Keywords:** difficult reintubation, difficult extubation, modified mallampati class, prone position, spine surgery, postoperative airway deterioration, airway edema

## Abstract

Background: Perioperative airway changes due to anesthesia and surgery could change a normal airway at induction to a risky airway at extubation.

Objectives: The objective is to evaluate primarily the degree of airway changes, as quantified by the modified Mallampati (MMP) class, after spine surgery in the prone position. Secondary to assess the time required for these changes to revert back to the preoperative state and their correlation with other demographic and surgical variables.

Methods: The present prospective observational study was conducted in a tertiary care hospital after ethical approval and trial registration. Fifty ASA I and II patients aged 18-65 years of both sex and undergoing spine surgery in prone positions were included. Supine MMP grade was observed preoperatively and at one, two, four, 24, and 48 hours postoperatively.

Statistical analysis: IBM SPSS version 22 (IBM Corp, Armonk, NY) was used.Mean values were compared using paired t-tests and medians by the Wilcoxon test. The Spearman correlation was used to assess a relationship. The time for recovery was analyzed by Kaplan-Meir analysis.

Results: An increase in MMP grade was observed at one hour postoperatively in 46 (92%) patients. Changes reverted back in 45 (98%) patients by 24 hours postoperatively. A weak positive correlation with age, weight, body mass index, duration of surgery, perioperative drop in hemoglobin, and a moderate positive correlation with fluid administered and estimated blood loss was recorded.

Conclusions: An increase in postoperative MMP occurs in the majority of patients undergoing prone position spine surgery which may persist up to 48 hours. So, more vigilance and caution are warranted should reintubation be needed postoperatively.

## Introduction

Safe extubation is crucial as nearly 30% of serious airway complications occur at extubation or recovery [1]. Several anatomical and physiological changes could change a normal airway at induction to risky at extubation. Possible determining factors for postoperative airway edema are prone or Trendelenburg position, inflammatory response, fluid administered, and blood loss [2,3].

Various studies evaluated the development of airway edema during surgeries or labor [4,5]. But very few had studied airway deterioration during prone position surgeries and their regression back to a preoperative state [3,6]. Thus, the present study was designed to investigate the degree of airway edema after prone position spine surgery, their regression time, and their correlation with other demographic and surgical variables.

## Materials and methods

After obtaining approval from the institutional ethics committee and registering with Clinical Trials Registry-India with reference number CTRI/2020/07/026746, the present observational study was conducted in tertiary care hospital from July 2020 to December 2020 in a prospective manner. Fifty patients of ASA I-II aged 18-65 years of either sex undergoing elective spine surgery in a prone position were included in the study. The exclusion criteria were pre-operative modified Mallampati (MMP) grade 4, pre-existing oropharyngeal pathology, inability to fully open mouth for assessment, severely limited neck mobility, history of difficult intubation, or difficult mask ventilation, cervical spine surgery, and patient refusal to participate in the study.

During the preoperative visit, the patient’s demographic data such as age, gender, height, weight, and body mass index (BMI), detailed clinical history, and complete general physical as well as systemic examination were done. Routine investigations like hemoglobin (Hb), bleeding time (BT), clotting time (CT), and complete urine examination were carried out in all patients. Any other relevant investigation like blood urea, serum creatinine, serum electrolytes, chest x-ray, and ECG were done as and when required. The purpose and protocol of the study were explained to all the patients and informed written consent to participate in the study was obtained. A standard airway assessment protocol was followed and observations were made with patients in the supine position by standing at the patient’s side preoperatively and at one, two, four, 24, and 48 hours postoperatively. For Mallampati class assessment, the patient’s head was placed on a 10-cm high pillow, mouth opened as wide as possible, tongue maximally protruded and without phonation and the observer assessed the airway by looking vertically downward [7]. All airway evaluations were performed by two senior anesthesiologists with at least five years of experience. All subjects received general anesthesia with the American Society of Anesthesiologists (ASA) standard monitoring. Proper padding of the eye and pressure points was done. The patient’s head was kept in a neutral position on a horseshoe-shaped soft sponge headrest (bolster). And a slight head-up tilt was used to keep the head above the level of the heart. Crystalloid in the form of Ringer Lactate was used for intraoperative fluid administration in all patients. Surgical parameters like duration of surgery, the total amount of intravenous fluids infused, intraoperative blood loss, and perioperative drop in hemoglobin were also recorded. Airway was reassessed in all patients at the end of surgery before extubation by direct laryngoscopy for visual inspection of the oral cavity edema and by cuff-leak test to look for subglottic edema that might preclude the extubation. The patients who required reintubation within 48 hours or were not extubated on the operation table were excluded from the analysis as it would not be possible to assess the degree of MMP change and the time for these airway changes to revert to baseline in such cases. Patients with other covariates that might result in increased airway edema like trauma during laryngoscopy (e.g., in anticipated and unanticipated difficult airway patients) and undergoing surgery that requires manipulations and instrumentation in the neck regions (e.g., cervical spine surgery) were also excluded from the study.

The sample size was calculated based on a study that reported the mean increase in MMP scores after surgery from the preoperative for three attending anesthesiologists [6]. Assuming these increases of 1±0.71, 1±0.75, and 0.8±0.85 values as a reference, the minimum required sample size at a 5% level of significance and 95% power was six, seven and 15, respectively. Initially, the sample size of 20 was proposed at the beginning of the study taking into consideration the dropout, but later on, we analyzed 50 patients due to the longer time frame of the study and the availability of such patients undergoing prone spine surgeries in our institution.

IBM SPSS version 22 (IBM Corp, Armonk, NY) was used for statistical analysis. For normally distributed quantitative parameters, mean values were compared; the change in the quantitative parameters, before and after the intervention was assessed by paired t-test (in case of two time periods). For non-normally distributed quantitative parameters, medians and interquartile range (IQR) were compared, and the change in the quantitative parameters, before and after the intervention was assessed by the Wilcoxon test. The Spearman correlation was used to determine if there was a relationship between two categorical variables. A p-value of <0.05 was considered statistically significant. The time for recovery among the entire population was analyzed by Kaplan-Meir analysis.

## Results

**Figure 1:**
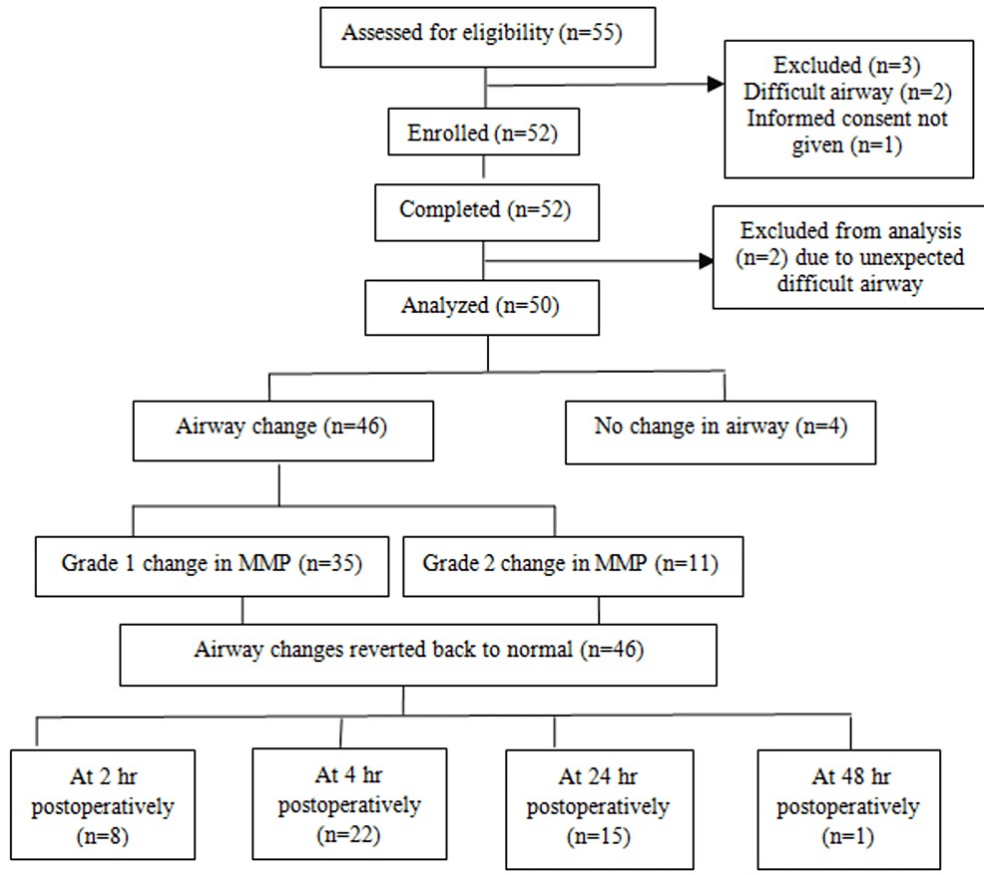
Consort study MMP - modified Mallampati Class

A total of 50 patients were analyzed for the study (Figure 1).

The demographic profile, baseline airway, and surgical parameters of the study population are shown in Table 1.

**Table 1:** Physical characteristics’ and surgical parameters of study population Values are in mean±SD or percentage

Characteristics	N=50	Range
Age(yr)	31.02±9.52	18-50
Gender male/female (%)	32/18(64/36%)	-
Height (cm)	161.88±6.93	152-172
Weight (kg)	64.28±10	40-90
BMI (kg/m^2^)	24.44±2.88	18-31.10
Baseline MMP 1/2/3 (%)	11/30/9 (22/60/18%)	-
Thyromental Distance (cm)	8.69 ± 0.81	7.5-10
Sternomental Distance (cm)	17.32 ± 1.77	13-20
Neck Circumference (cm)	32.38 ± 3.26	28-40
Duration of Surgery (min)	145.8 ± 36.17	90-220
Intravenous Fluids (mL)	1265 ± 346.74	900-2,200
Estimated Blood Loss (mL)	316.6 ± 117.47	150-500
Hemoglobin drop (g/dL)	0.31±0.2366	0-1

**Figure 2:**
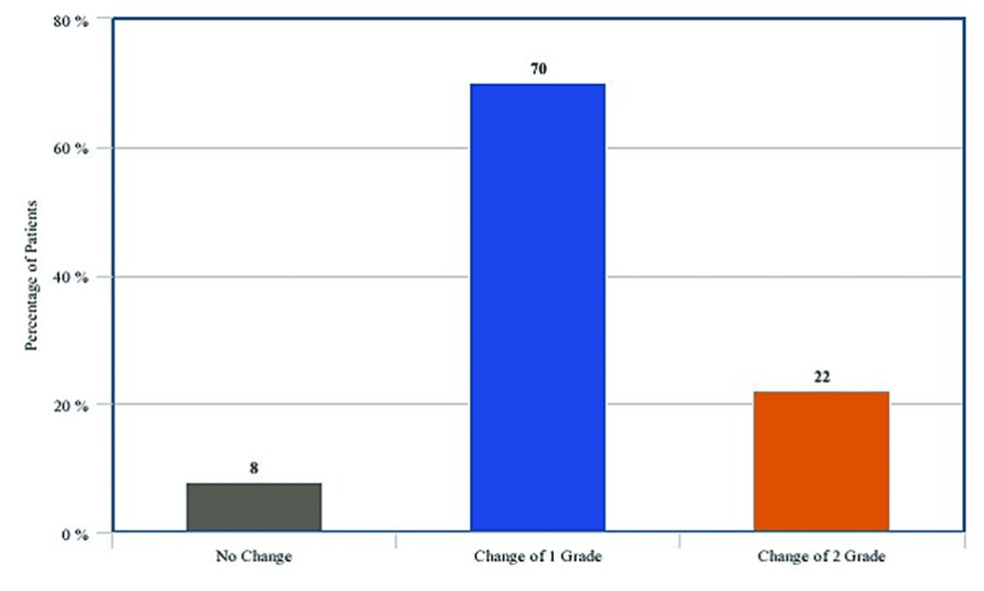
Bar diagram depicting the change in modified Mallampati class and degree of change (N=50)

On baseline airway assessment, MMP score was one, two, and three in 22%, 60%, and 18% of patients, respectively. An increase in MMP grade was observed in 46 (92%) patients while four (8%) patients showed no change (Figure 2).

**Figure 3:**
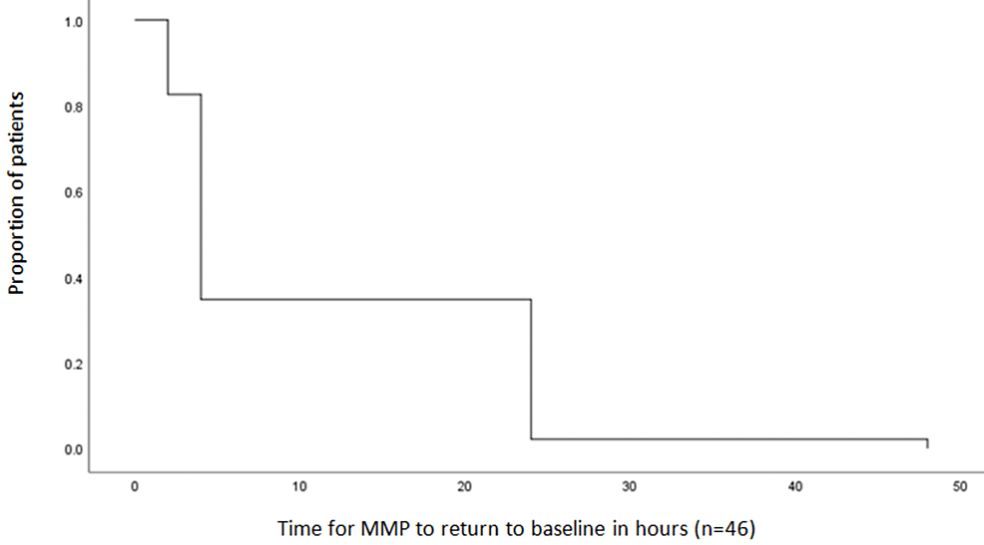
Kaplan-Meier curve showing average time for modified Mallampati class recovery in study population (N=46)

Thirty-five patients (70%) had a change of one grade and 11(22%) patients had a change of two grades in MMP. All these patients who had deterioration in airway, maximum change was observed at one hour postoperatively. These patients were then followed up to see how long this change in MMP score persisted. Changes in MMP grades reverted back to their preoperative state by two hours in eight (17.4%), by four hours in 22 (47.8%), by 24 hours in 15 (32.6%) and by 48 hours postoperatively in one (2.2%) patient (Figure 3).

In 98% cases, MMP class change resumed baseline status in first 24 hours. Only one patient had higher than baseline MMP score by one grade at 24 hours postoperatively, that too reverted back to preoperative state at 48 hours. So, none of the patient had shown persistence of airway change after 48 hours postoperatively. Comparison of preoperative and postoperative median MMP score is shown in Table 2.

**Table 2:** Comparison of median Mallampati score at different time periods (N=50) hr-hours, * p<0.05 Statistically Significant, # p>0.05 Statistically Insignificant

Time	Mallampati score Median (IQR)	(Wilcoxon signed test) p value
Preoperative	2 (2, 2)	-
Postoperative 1 hr	3 (3, 3)	<0.001*
Postoperative 2 hr	3 (2, 3)	<0.001*
Postoperative 4 hr	2 (2, 3)	<0.001*
Postoperative 24 hr	2 (2, 2)	0.317^#^
Postoperative 48 hr	2 (2, 2)	1.000^#^

The difference was statistically significant at postoperative one, two and four hours (p-value<0.05). The difference thereafter was statistically insignificant (p-value>0.05) depicting that by 24 hours postoperatively most of the airway changes reversed back to baseline. A weak positive correlation of degree of change in MMP with age, weight, BMI (body mass index), duration of surgery and perioperative drop in hemoglobin was observed (Table 3). A moderate positive correlation of maximum MMP change was recorded with intravenous fluid administered and estimated blood loss (Table 3). None of our patient had any airway related or respiratory complication at extubation.

**Table 3:** Correlation between change in modified Mallampati class and different demographic, surgical parameters *Weak or no relation - rs 0.4 or less #moderate relation - rs = 0.5-0.7 **Strong correlation - rs = >0.7

	Spearman rank correlation (r_s_) [P-value]
Age	0.431* [0.002]
Weight (in kg)	0.355* [0.011]
Height (in cm)	0.109* [0.451]
BMI (Kg/m2)	0.289* [0.042]
Duration of Surgery (min)	0.275* [0.054]
Intravenous Fluids (ml)	0.546^#^ [0.000]
Hemoglobin drop	0.3647* [0.009]
Blood Loss(ml)	0.511^#^ [<0.001]

## Discussion

Perioperative airway deterioration may result in increased chances of airway mishaps following tracheal extubation in patients undergoing prone position surgeries [2]. It may result due to airway trauma at laryngoscopy and intubation, surgical stress syndrome, or other non-surgical factors. Important determining factors for postoperative edema formation as determined by the Difficult Airway Society were prone or Trendelenburg positioning, prolonged surgical duration, and fluid overload [2]. In a prone position, increased intrathoracic and intraabdominal pressure, non-neutral, flexed and lower than heart position of the head, presence of endotracheal tube in oropharynx with excessive neck flexion, may affect the venous drainage from lingual and pharyngeal veins and thereby can result in tongue and oropharyngeal swelling [8,9]. This prolonged venous stasis along with kinking and trauma to vascular intima and hypercoagulation state due to surgical stress together completes the Virchow triad leading to an increased probability of venous thrombosis causing more local swelling [8]. Hence, pressure and ischemia are the root cause of mucosal edema, which can progress slowly and presents as airway obstruction at extubation [10]. As airway trauma resulting from repeated attempts at laryngoscopy can also contribute to oropharyngeal swelling, so we did not include the difficult airway patients in our study [8].

Although some degree of airway edema develops in nearly all patients postoperatively, very few of them cause problems in reintubation or at extubation. We also observed airway deterioration in most of our patients (92%) in the immediate postoperative period post-extubation but we could not assess the actual increase in intubation difficulty as none of our patients required reintubation in the postoperative period. Similar airway changes were observed by various authors postoperatively and during labor/delivery although the incidence varies [3-6,11]. There is a discrepancy among different studies regarding the time for maximum airway changes seen. In the present study, maximum airway deterioration was seen at the first assessment, i.e., one hour postoperatively. Similar to our findings, some researchers observed maximum airway deterioration in the immediate postoperative period [5,6]. While in another study, maximum postoperative airway edema was recorded at four hours in 86.4% of patients and eight hours in rest 13.6% of patients [3].

As airway edema is usually transient and self-limiting, we further assessed the airway postoperatively at different time points to follow its trend. All our patients were safely extubated at the end of surgery after doing airway reassessment. We found that in most of the patients MMP changes resumed their baseline status in the first 24 hours, and none of our patients had persistent airway edema beyond 48 hours. Only two other studies, one in prone position nephrolithotomy surgery and another in obstetric patients during labor, followed the change in MMP postoperatively and post-labor. Contrary to ours, both recorded persistence of airway changes beyond 48 hours (in 3% of patients postoperatively and in 21% of parturients post-labor) [3,4]. But both of these authors could not identify any factor that could predict the persistence of airway changes after 48 hours. Other researchers in their studies did not follow the persistence of these airway changes [5,6].

To identify the other predictive factors for postoperative airway edema development, we also assessed the correlation between the degree of MMP change and various patient related and surgical variables. We found a weak correlation between age and degree of MMP change postoperatively (Table 3). Not many studies had assessed the effect of age on postoperative airway changes and the few that had studied, found no such relation [3]. Possible hypothesis for this relation seen is related to the observed higher surgery-induced IL-6 levels in elderly patients [12]. As the surgical stress induced inflammation is one of the contributing factors for the airway edema development, so age could be a contributing factor for airway deterioration postoperatively.

Most of the studies did not find any correlation between weight and postoperative or post labor airway deterioration [3,5,11]. A weak correlation with BMI was recorded in present study (Table 3). None of our patients had BMI>30 kg/m^2^, but some patients were overweight with BMI between 25 and 30 kg/m^2^. Obesity being a part of metabolic syndrome that itself is associated with a variety of morbid conditions like exaggerated inflammatory response and a possible link has been proposed between visceral obesity and increase in proinflammatory cytokines such as TNF-alpha, IL-6 along with increased oxidative stress [13]. So, this proinflammatory state along with increase in intrathoracic pressure in prone position could be a possible explanation for the increase in development of airway edema with increase in body weight.

As the duration of surgery increases, the length of the time for elevated venous pressure in the head and neck, fluid shifts, capillary leak, development of interstitial edema formation, and systemic inflammation also increases. Although most of the authors did not find any correlation among duration of surgery/labor and postoperative/post labor fluid accumulation and airway deterioration [3-4,6,14]. But a weak correlation between postoperative MMP change and duration of surgery was observed in our study (Table 3). Similarly a previous research, observed a weak correlation between surgical duration and airway dimensions’ changes postoperatively [5]. Also, a positive correlation between duration of second stage of labor and decrease in pharyngeal volume had been reported [11]. However, no strong association was observed. This could be because of the limited duration of surgery in our study (145.8 ± 36.17 min, ranging from 90 to 220 min, Table 1). Also, it has been postulated that in comparison to duration of surgery, intensity of surgical stress response is a better predictor of fluid retention.

In the present study, a moderate positive correlation was recorded between degree of change in MMP score and intravenous fluids (mL) administered intraoperatively (Table 3). In literature search, results are controversial as only one researcher found a weak correlation [5]. Contrarily, others did not observe any correlation between degree of MMP grade change and volume of crystalloid administered [3-4,6,11]. Difficult Airway Society (2012) identified fluid overload as possible contributor to airway edema [2]. As fluid overload can favor the Starling equation toward exacerbating mucosal edema as a result of increased transendothelial fluid exchange at capillaries resulting from decreased oncotic pressure [15]. In the presence of increased capillary hydrostatic pressure as occurs with venous obstruction and gravity induced elevation in dependent venous pressure in prone position, it results in more edema formation [15]. Similar mechanism also seen when displaced fluid from the lower limbs on inflation of antishock trousers can lead to increase in neck circumference [16]. Similar findings were observed in patients with congestive heart failure (CHF) and renal insufficiency at night and is also the probable cause of airway changes due to fluid retention in pregnancy and labor [17].

We observed a moderate relation of postoperative MMP change with blood loss and a weak correlation with postoperative drop in hemoglobin but none of our patient had excessive blood loss intraoperatively and no strong association was observed (Table 3). It has been observed that fluid resuscitation done to treat massive bleeding caused a rapid deterioration in Mallampati class postoperatively [11,18]. Proposed mechanism is the decrease in the colloid osmotic pressure that might contribute to the increased tissue inflammation and development of airway edema [11].

Therefore, MMP score can change postoperatively in prone position surgery. Hence, for the safe conduct of anesthesia and tracheal extubation, due safety measures must be ensured intraoperatively and prior to extubation.

Limitations of the study

In the early postoperative MMP evaluation, there were chances of getting subjective error due to inability of patients to open mouth maximally on command. To counteract this error, patients were trained preoperatively to open mouth as wide as possible and protrude tongue fully without phonation. Also, to prevent effect of posture on MMP scores from interfering with the comparison, all assessments were done in similar position (supine position). Although surgical factors like duration of surgery, intraoperative fluid administration, estimated blood loss, perioperative hemoglobin drop and demographic factors like age, weight, BMI depicted some degree of positive relation with postoperative airway changes, no strong correlation was observed. The sample size may not be adequate for evaluating the correlation of these parameters and the correlation may have occurred merely by chance. So, further larger sample size studies are required before we can be really sure that a true cause effect relationship is there among these factors and postoperative airway edema formation.

## Conclusions

The present study shows that there is at least one grade of increase in the MMP score in the majority of patients undergoing spine surgery in a prone position postoperatively. This increase in the MMP score generally reverts back to baseline in the first 24 hours postoperatively but may persist even up to 48 hours in some patients.

The degree of airway change is weakly correlated with the duration of surgery but moderately influenced by the amount of intravenous fluids used and estimated blood loss. Such worsening of MMP score and its persistence in the postoperative period may increase the chances of encountering difficulty at extubation and reintubation (if required), especially in the presence of other pre-existing risk factors for difficult airway. So, more vigilance and caution are warranted should reintubation be needed in the postoperative period after surgery in the prone position.
